# T118. IMPACT OF DYSFUNCTIONAL METACOGNITIONS AND WORRY ON DEVELOPMENT OF PARANOIA: A 1-YEAR LONGITUDINAL STUDY IN A NON-CLINICAL SAMPLE

**DOI:** 10.1093/schbul/sby016.394

**Published:** 2018-04-01

**Authors:** Xiaoqi Sun, Suzanne So, Raymond Chan, Chui-de Chiu, Patrick Leung

**Affiliations:** 1 The Chinese University of Hong Kong; 2 Institute of Psychology, Chinese Academy of Sciences

## Abstract

**Background:**

A worry thinking style has been identified as one of the proximal causal factors for paranoia (Freeman & Garety, 2014). This argument has been supported by the finding that patients with paranoia worry as much as patients with generalized anxiety disorder, and that worry predicts paranoia in non-clinical individuals. Wells (1995) argued that it is when metacognitions about worry (i.e. beliefs about worry and meta-worry) exaggerate worrying that anxiety disorders emerge. It was not clear how metacognitions interact with trait worry in the development of non-clinical paranoia.

**Aims:**

To examine the predictive effect of dysfunctional metacognitions and trait worry on change in paranoia over one year within a large university sample.

**Methods:**

An online survey encompassing measures of metacognitions, trait worry, and paranoia was conducted at baseline (valid N=2291) and one year (N=1746). A series of longitudinal structural equation models were tested, with baseline level of metacognitions as latent variable, baseline trait worry and paranoia at both time points as observed variables. Model fit indices were compared across models (CTI, RMSEA, AIC, BIC).

**Results:**

A final trimmed model with the best goodness-of-fit (χ^2^=82.78, p<.001, CFI=0.99, RMSEA=0.069) suggested that dysfunctional metacognitions contributed to paranoia at 1-year follow-up, both directly (β=0.21, p<.001) and via baseline paranoia (β=0.09, p=.001). Trait worry at baseline did not predict paranoia at either time point.

**Discussion:**

Our results indicated a critical role of dysfunctional metacognitions in paranoid ideation both concurrently and prospectively. Future interventions may focus more on modifying beliefs and worry about worry.

